# Identification of Deceased Soldiers

**Published:** 1864-11

**Authors:** Josiah Curtis

**Affiliations:** Surgeon U. S. V., Acting Medical Director; Headquarters Department of the Ohio, Medical Director’s Department, Knoxville, Tenn.


### IDENTIFICATION OF DECEASED SOLDIERS.

HEADQUARTERS DEPARTMENT OF THE OHIO,
MEDICAL DIRECTOR'S DEPARTMENT,
KNOXVILLE, TENN., *June* 1, 1864.

[*Circular Letter.*]

Upon the death of a soldier in this military department—whether in hospital or in the field—the Chaplain—wherever one is on duty, and in all other cases the Surgeon, is instructed, whenever practicable, to cause the *name, rank, company, regiment, age, date and cause of death, last place of residence*, and any other items deemed of importance relating to the deceased, to be legibly written upon white paper, with ink, and to place this record in a bottle, to be well corked, and deposited in the coffin, at the foot of the body, before burial.

JOSIAH CURTIS,
Surgeon U. S. V., Acting Medical Director.

---

Messrs. Lippincott & Co. of Philadelphia, have in press a new edition of Wood & Bache's United States Dispensary.

